# Development and Psychometric Evaluation of the Chinese Feeding Difficulty Index (Ch-FDI) for People with Dementia

**DOI:** 10.1371/journal.pone.0133716

**Published:** 2015-07-21

**Authors:** Megan F. Liu, Nae-Fang Miao, I-Hui Chen, Yen-Kuang Lin, Mu-Hsing Ho, Beverly L. Roberts, Chia-Chi Chang

**Affiliations:** 1 School of Gerontology Health Management, College of Nursing, Taipei Medical University, Taipei, Taiwan; 2 School of Nursing, College of Nursing, Taipei Medical University, Taipei, Taiwan; 3 Department of Nursing, College of Medicine and Health Science, Asia University, Taichung City, Taiwan; 4 Biostatistics Research Center, Taipei Medical University, Taipei, Taiwan; 5 Graduate institute of Nursing, College of Nursing, Taipei Medical University, Taipei, Taiwan; 6 School of Nursing, University of Florida, Gainesville, Florida, United States of America; Cardiff University, UNITED KINGDOM

## Abstract

**Aims:**

To develop and evaluate the psychometric properties of a Chinese Feeding Difficulty Index (Ch-FDI) which assesses feeding difficulties in people with dementia (PwD).

**Research Design and Method:**

Scale items were developed using literature review based on Model of Feeding Difficulty. Content validity was evaluated and items were modified by expert panel. Following translation and back-translation, the Ch-FDI was piloted on residents with dementia. The reliability was tested by inter-rater reliability and test-retest reliability. Internal reliability was established by calculating Cronbach's α coefficient. The concurrent validity was evaluated by correlating with similar scale, the Edinburgh Feeding Evaluation in Dementia (EdFED). The exploratory factor analysis (EFA) with varimax rotation and parallel analysis (PA) was performed to test construct validity.

**Method:**

Participants were recruited from long-term care facilities in Taiwan. A total of 213 residents with dementia participated in this study during May, 2010 to February, 2011.

**Results:**

Content validation, translation and psychometric testing were completed on the 19 items of the Ch-FDI. The translated scale was piloted on 213 residents with dementia of feeding difficulty who were recruited from eight long-term care facilities in Taiwan. The reliability was supported by the internal consistency of Cronbach's α of 0.68 and a test-retest coefficient of 0.85. The content validity, face validity, concurrent validity, and construct validity were used.

**Conclusions:**

The Ch-FDI is a newly developed scale with fair psychometric properties aimed to measure feeding difficulties among residents with dementia in long-term care facilities in Taiwan. Using this reliable and valid tool can help healthcare providers to assess feeding problems of PwD and provide feeding assistance in order to promote quality of care during mealtime in long-term care facilities.

## Introduction

In recent years, care of people with dementia (PwD) has become the focus of medical care, and feeding difficulty is a common problem among PwD. According to previous studies, the prevalence rate of feeding difficulty among PwD was around 60% [1,2]. Depending on their individual level of impairment, they may clamp their mouth shut, spill and pool food in their mouth, delay to swallow, and refuse to eat. Serious feeding difficulties may be associated with weight loss, dehydration, malnutrition, and death [3]. Factors associated with feeding problems of PwD are complicated including physical functions, psychological and social factors, the dining environment, and cultural issues [4]. Physical functions such as impaired motor skills, chemosensory changes, olfactory and visual impairment, and dental or oral health are related to the feeding and nutritional problems of PwD. Psychological factors, especially a diagnosis of depression, are associated with food intake in people with dementia. Moreover, social interactions between caregivers and PwD during mealtime also contribute to the complexity of feeding difficulties among them. In addition, the dining environment is also an important part of feeding process. Feeding PwD in a quiet, relaxed atmosphere dining room has shown to increase food intake and mealtime socialization, and decrease late-day agitation. Cultural issues, such as lack of ethnic foods, unable to meet special eating habits, and self-perception of older adults are related to the eating ability of PwD [3].

Feeding difficulties are specific behaviors elicited while the caregiver is feeding the individual. These difficulties include problems the caregiver encounters in getting food into the mouth and in assisting the individual to overcome or compensate for problems with chewing and swallowing. In addition, feeding difficulties also include difficulty in assisting the individual in initiating feeding and maintaining attention to the feeding task [5]. As a result, the feeding difficulty among PwD could indicate any difficulty or problem occurred during mealtime and associate with physical, cognitive, behavioral, social, environmental and cultural factors. Eating might be the only daily activity function that older adults still keep in their later life [6] and being able to regain after training [7]. Eating is not only related to physical health, but also to quality of life. It is needed to develop an appropriate instrument to assess eating difficulties among PwD that can help nurses to identify eating problems and further provide suitable interventions [8]. Therefore, an instrument that can measure eating difficulties of PwD and has sufficient sensitivity to detect changes in eating abilities is essential.

The Edinburgh Feeding Evaluation in Dementia (EdFED) scale was developed by Watson and Deary and its purpose is to help clinicians to determine the level of feeding assistance based on observed eating and feeding problems [9–13]. However, this instrument does not capture all feeding problems, such as difficulty getting food into the mouth, chewing, swallowing, or paying attention to the task of eating, as well as feeding difficulties related to social, cultural, and environmental factors or interactions with nursing assistants [5,14,15]. Additionally, the culture, eating habits, diet, and healthcare services and practices differ between Western and Eastern countries. Consequently, it is necessary to develop a suitable instrument with valid psychometric properties in order to evaluate feeding problems and consequently to provide proper interventions. The principal investigator (CCC) has identified five general types of feeding difficulties involving the following tasks: initiating the feeding, maintaining attention, getting food into the mouth, chewing food, and swallowing food [5]. Additionally, we described several specific manifestations and the observable behavior within each of those general areas [15]. As there was no suitable assessment tool available, a new scale, Feeding Difficulty Index (FDI), was developed base on the principal investigator (CCC) previous work. The aims of this study were as follow:

1. To develop and identify items of FDI to assess feeding difficulties among people with dementia
2. To translate the items into Chinese
3. To pilot the developed scale in a primary care population to assess acceptability and feasibility of administering the questionnaire to people with dementia
4. To examine the psychometric properties of the Chinese Feeding Difficulty Index

## Methods

The principal investigator explained the content of the written consent form to family or guardians of eligible participants. Every eligible participant had written proxy consent from their family or guardian. The study and the consent procedure were approved by the Institutional Review Board from the Human Subject Committee of Taipei Medical University (no. P970331).

### Instrument Development

The original Feeding Difficulty Index (FDI) consists of 24 items and was identified and derived base on the five general types of feeding difficulties involving the following tasks: initiating the feeding, maintaining attention, getting food into the mouth, chewing food, and swallowing food [5]. Then the FDI was sent to eight experts to review for its content validity. The eight experts specialize in the field of neurology, psychiatry, gerontology, and rehabilitative medicine; while three of experts also expertise in instrument development and psychometric testing. The FDI was evaluated for its relevance, applicability, representativeness, specificity, and clarity and each item from all five aspects was rated from 1 “very low” to 5 “very high”. The values of Content Validity Index (CVI) for relevance, applicability, representativeness, specificity, and clarity were 88.9%, 88.2%, 88.2%, 87.5%, 92.3%, and 96.5%, respectively. Five items were recommended to be eliminated because of the CVI scoring less than 80%. Due to inconsistence with the definition of feeding difficulties, difficulty in observation, and similarity with other items; seven items were reworded in accordance with experts’ recommendations. Consequently the FDI was modified to 19 items and was administered and rated during the entire meal time of PwD by observing the number of times a behavior arose with each food offering. Each item was recorded based on the number of behaviors as 0 times (0 point), 1 or 2 times (1 point), 3 to 5 times (2 points), and 6 or more times (3 points). Scores range from 0 to 57 with higher scores indicating greater feeding difficulties (S1 File). For the purpose of developing the Chinese version of the FDI, the instrument was first translated into Chinese by the principal investigator (CCC) and then back translation into English by another investigator (MFL) to assess for translational equivalence. Both investigators are bilingual with previous experience in questionnaire translation and development. With all nonequivalent items, wordings were modified to enhance the translational equivalence in compliance with the original English version by both investigators.

### Pilot psychometric testing of the Ch-FDI

The 19-item Chinese Feeding Difficulty Index (Ch-FDI) was pilot-tested on PwD who reside in long-term care facilities in Taiwan. PwD were recruited when meeting the following criteria: 1) aged ≥60 years; 2) receiving oral feedings; and 3) identified by the nursing assistants as having feeding difficulties and needing assistance during mealtimes. On the other hand, PwD who were in a coma or using artificial feeding were excluded. All eligible participants were recruited continuously until the sample size was reached. Based on the subject to item ratio of 10:1, a sample size of 190 participants was required as Ch-FDI consists of 19 items [16]. Furthermore, a sample size of at least 200 might be needed for a more conservative estimation [17].

### Data Analysis

**Fig 1:**
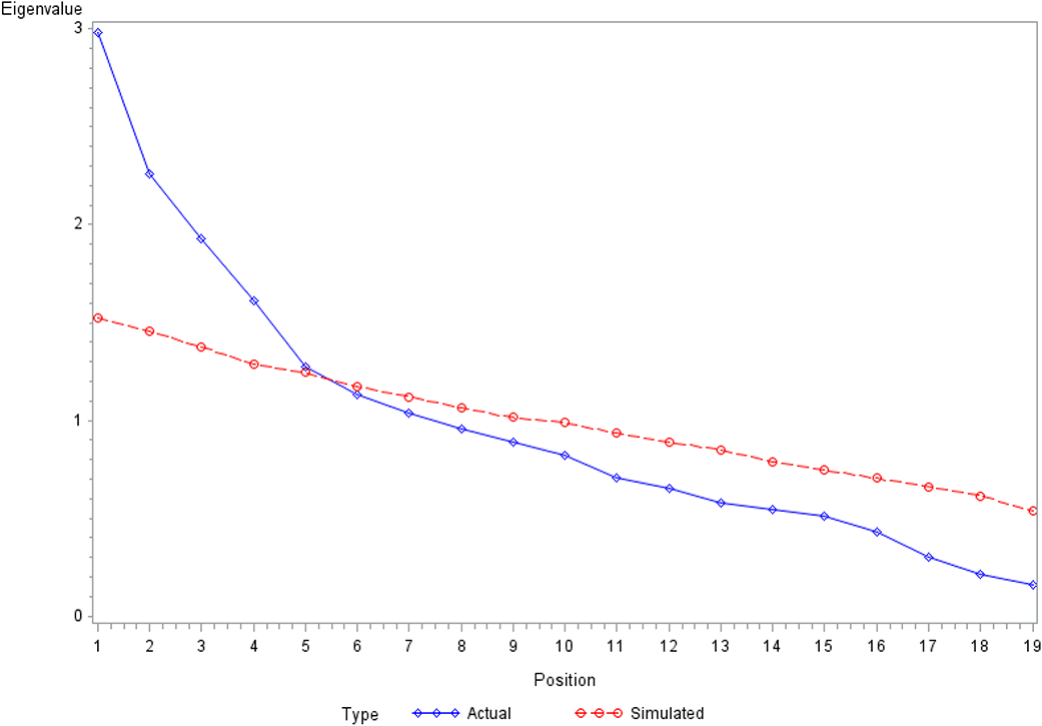
Median simulated eigenvalues of parallel analysis.

All data were analyzed using SAS statistical software packages, Version 9.4. Descriptive statistics were performed including means, standard deviations (SDs), frequencies, and percentages. The reliability and validity of the Ch-FDI were evaluated as follow. The internal consistency was established by calculating Cronbach's alpha coefficient and it ranges between 0 to1. A Cronbach's *α* coefficient ≧ 0.6 was used as the cut-off point to indicate sufficient internal reliability [16]. The factor extraction was using the principal components analysis with varimax rotation. The Kaiser-Meyer-Olkin (KMO) measure of sampling adequacy (using a cut-off of 0.5), and Barlett’s Test of Sphericity (using a cut-off *p*<0.001) was used to ensure the appropriateness of data set for EFA [16]. Based on a sample size of 200, a factor loading of 0.384 of EFA should be adequate [18]. In this study we took a factor loading of >.4 into consideration and then decide whether to keep or discard each item. Parallel analysis (PA) was then used to assist with determine the number of factors to extract in EFA [19] (Fig 1).

## Results

### Inter-rater reliability and test-retest reliability

First, six observers which compose of three research assistants, two nurses, and one nursing assistant were trained to use the Ch-FDI. Later they observed ten PwD simultaneously in order to determine the inter-rater reliability. The mean agreement rate of the six observers on the ten PwD was 0.87and the average agreement rate of each items ranged between 0.55 and 0.98.

Another ten PwD with feeding difficulties were recruited to be observed two times with an interval of two weeks to assess the test-retest reliability. The correlation coefficient between the pre-test and post-test of the Ch-FDI was 0.85 (*p*<0.01) and the paired *t*-test showed that there was no significant difference between the two tests (*t* = 0.355, *p*≈0.73).

### Participant characteristics

A total of 213 PwD were included in the analysis. Over half of the study sample were female (57.3%), Minnanese (60.0%), widowed (54.2%) with an average age of 82.6 years old (SD = 6.9). The mean length of time with a diagnosis of dementia was 2.5years (SD = 2.4). The mean scores of the Mini-Mental State Examination (MMSE), Activities of Daily Living (ADL), and Geriatric Depression Scales-Short form (GDS-S) were 8.9 (SD = 8.2), 40.4 (SD = 33.7), and 3.5 (SD = 2.9), respectively (see Table 1).

**Table 1:** Characteristics of subjects (*N* = 213).

Variable	Mean (standard deviation)/*n* (%)
**Gender**	
Male	91 (42.7)
Female	122 (57.3)
**Age (years)**	82.7 (6.7)
**Duration of dementia (years)**	2.5 (2.4)
**Ethnicity**	
Minnanese (Taiwanese)	129 (60.6)
Hakkanese	8 (3.8)
Mainlander	74 (34.7)
Others	2 (0.9)
**Education**	
Illiterate	53 (24.9)
Informal education	10 (4.7)
Elementary	65 (30.5)
Junior-high	23 (10.8)
Senior-high	29 (13.6)
College	32 (15.0)
Masters or above	1 (0.5)
**Marital Status**	
Single	16 (7.5)
Married	72 (34.0)
Divorced	9 (4.2)
Widowed	115 (54.2)
**MMSE** ^a^	8.9 (8.2)
**ADL**	40.4 (33.7)
**GDS-S** ^b^	3.5 (2.9)
**BMI (kg/m** ^**2**^ **)**	21.5 (3.8)

^a^ MMSE, Mini-Mental Status Examination; ADL, Independence in Activities of Daily Living

^b^ GDS-S, Geriatric Depression Scale- Short form; BMI, body-mass index.

Around 60% of subjects had verbal or non-verbal communication during their mealtime. The mean eating time and food intake were 20.6 minutes (SD = 15.1) and 506.2 gram (SD = 150.5). The mean scores of the EdFED and Ch-FDI were 4.9 (SD = 3.9) and 4.0 (SD = 3.5). The mean BMI was 21.5 kg/m^2^ (SD = 3.8), and less than a quarter of PwD’ BMI (21.6%) were less than 18.5, which is indicated as malnutrition, defined by the World Health Organization.

### Internal consistency

The internal consistency was established by calculating Cronbach's *α* coefficient, which was 0.61, indicating fair internal consistency of the Ch-FDI.

### Concurrent validity

The concurrent validity was evaluated by correlating the Ch-FDI with the EdFED. The Ch- FDI score was significantly correlated with the EdFED score (*r* = 0.6, *p*<0.01, *n* = 213). Additionally, the Ch-FDI score was significantly correlated with MMSE (r = -0.2, p<0.01, n = 213), ADL (r = -0.181, p<0.01, n = 213), BMI (*r* = -0.137, *p*<0.05, *n* = 213), and eating time (*r =* 0.391, *p<*0.01, *n* = 213).

### Construct validity

EFA was used to test construct validity, and rotation method was varimax with Kaiser Normalization. PA was then used to assist with determine the number of factors to extract in EFA [19]. Together with the result of PA and taking adequate factor loading (>.4) of EFA into consideration, three items including item 7, 8 and 10 were deleted and thus four factors including 16 items were finalized. Four factors among the scale items could explain 52.6% of the variance of the Ch-FDI. The KMO measure was 0.7 indication sampling adequacy. Sufficient variability in the data was confirmed by Bartlett’s Test of Sphericity (*p* <0.001) confirming the validity of data available for EFA [16]. The four factors were renamed as 1) difficulties with getting food, 2) distraction, 3) food refusal, and 4) motor difficulties, Cronbach's *α* values for the four factors were 0.65, 0.71, 0.48, and 0.41, respectively (Table 2).

**Table 2:** The factor loadings of exploratory factor analysis.

Rotated Component Matrix ^a^		Factor loadings				Cumulative Variance (%) ^b^
Description of the Factors and Items ^c^		1	2	3	4	
**Factor 1: Difficulties with getting food**						15.055
6	Does not open mouth or bites the utensils when food is offered	**.764**	.033	.127	-.246	
4	Turns head away or tilts head backward	**.610**	.003	.212	-.315	
17	Once food is in the mouth, food dribbles out from the mouth	**.578**	-.004	-.001	.317	
19	Chokes or gags on food	**.543**	-.064	-.094	.140	
5	Spits out the food	**.541**	-.083	-.035	.132	
18	Continuously chews food or holds in mouth but does not initiate swallowing	**.530**	.361	.101	-.022	
**Factor 2: Distraction**						29.002
11	Discontinues eating for over 1 minute	.124	**.874**	.061	.103	
9	Does not start to eat for at least 1 minute when invited to do so	.193	**.821**	-.003	.088	
12	Distracted from eating by talking, looking around, or watching TV	-.134	**.628**	-.051	-.028	
13	Plays with food: does something with food but not eating it	-.104	**.494**	-.005	-.036	
**Factor 3: Food refusal**						42.573
2	Negative behavior toward feeder: pushes, hits, kicks, or throws objects at feeder	-.015	.000	**.921**	.064	
3	Inappropriate verbal statements toward feeder	-.107	-.029	**.884**	.045	
1	Pushes or resists food offered by hand	.335	.029	**.649**	-.176	
**Factor 4: Motor difficulties**						52.632
14	Unable to successfully pick up food with utensil	.130	.079	.006	**.693**	
15	Once when food is on an eating utensil, unable to get food effectively into the mouth	-.133	-.052	-.139	**.622**	
16	Uses hand to feed self	.098	.040	.109	**.622**	
Cronbach’s α		0.65	0.71	0.48	0.41	

^a^ Extraction method: Principal Component Analysis. Rotation method: Varimax with Kaiser Normalization.

^b^ Cumulative variance explained after rotation.

^c^ Removed items: 7. Leaves the table 8. Cannot sit still: slipping or twisting body affecting eating. 10. Becomes drowsy or falls asleep.

## Discussion

The Ch-FDI is a newly developed scale and had fair psychometric properties for measuring feeding difficulties in PwD in long-term care facilities. Its reliability was supported by fair internal consistency as indicated by Cronbach's *α* and a good test-retest coefficient. Moreover, the measure’s validity was supported by the content validity, concurrent validity, and construct validity. The Ch-FDI was related to the EdFED scale with a correlation of 0.6, and yet the Ch-FDI did capture some different aspects of feeding difficulties in residents with dementia.

According to the factor analysis, four factors were extracted including difficulties with getting food, distraction, food refusal, and motor difficulties. Comparing to the five dimensions of Model of Feeding Difficulty, the problem behaviors in the factor 2 (distraction) might result in difficulty initiating and maintaining the feeding tasks. Two items were removed in the aspect of problem behaviors during the process of establishing adequate construct validity. These two items had low factor loading and low frequency (Item 7. Leaves the table and item 8. Cannot sit still: slipping or twisting body affecting eating.). Possible explanation may be that Taiwan’s physical restraints rate are high in comparison with other countries [20], especially at mealtimes. Nurses and nursing assistants in Taiwan often use restraining belt, sheet, seat-belt, and mitt to restraint resident’s movement [20, 21]. Mealtime phyiscal restraints are to prevent residents with dementia to leave their seats or to maintain their posture during mealtime while some of the residents might slip or twist their body when they are eating. The factor 1 (difficulties with getting food), 3 (food refusal), and 4 (motor difficulties) might be related to the dimension of difficulty getting food into the mouth. Regarding the difficulty in chewing and swallowing food dimensions in Model of Feeding Difficulty, problem behaviors due to chewing or swallowing are difficulty to be observed during the feeding process.

Feeding difficulties are associated with multiple factors, and dementia may not be the only cause to feeding problems but comorbid with other illnesses. Social and physical environments may be the reasons for feeding problems in PwD in long-term care facilities [22]. According to the study results, the feeding difficulty is correlated to cognition, physical function, depression, and nutritional status which are similar to the literature [1, 4, 5, 15]. Regarding items of the Ch-FDI, nursing assistants had difficulty in differentiating items 15 and 17 (S1 File), and these may be modified in the future.

The Ch-FDI was implemented by observing mealtimes and captured all problems during the eating process. Eating time was the major factor in feeding difficulties among residents with dementia. According to the results, the feeding difficulty is significantly correlated to eating time. A longer eating time might indicate a higher level of feeding difficulties among residents with dementia. Nurses and nursing assistants are the major caregivers for residents in long-term care facilities. Thus, it is important to educate nurses and nursing assistants to observe feeding problems among residents in long-term care facilities in order to provide adequate assistance during dining and to further prevent malnutrition. Moreover, nurses and nursing assistants need to pay greater attention to residents who have longer eating times. Training/educational programs were found to decrease feeding difficulties in elderly with dementia [4].

The dining environment was found to be an important part of the feeding process [23]. According to our observations, one possible explanation might be that kitchen services within long-term care facilities in Taiwan cannot satisfy residents’ needs. For instance, almost all of the long-term care facilities in Taiwan don’t have 24 hour open kitchen or cafeteria which could serve food or meals according to residents’ habit and to provide snacks or finger food between meals or at night based on residents’ needs. Consequently, residents could not follow their life-long dinning habit and need to follow the dinning schedule in the long-term care facilities which may trigger aggressive behaviors during the mealtime among residents with dementia [24]. Another possible reason of environmental factors affecting feeding difficulties was possibly the sundown syndrome among residents with dementia. Cognitive impairment may be short-term memory deficits, which interfere with eating because it causes the patient to forget the task at hand or become easily distracted due to environmental factors. A relaxed and quite dining environment that is not crowded might minimize residents with dementia becoming distracted by environmental factors [5,15]. Interactions with caregivers play an important role during mealtimes. Enhancing interactions and changing the context of meals are two modalities to influence mealtime outcomes for residents with dementia [25].

## Limitations

There are several limitations in this study. First, the EdFED is the only scale to compare with the newly developed scale; other instrument could be investigated in comparison with Ch-FDI in the future. In addition, a larger sample size may be needed on the basis of the low Cronbach’s *α* factor. Thirdly, the Ch-FDI is to assess the feeding problems by meal time observation, which might take a longer period of time to complete the scale as a result of the eating time. Lastly, a longitudinal study with multiple follow-ups will be needed to fully understand the change of feeding difficulties over time.

## Conclusions

A reliable and valid tool can help health providers to assess feeding problems of PwD and further provide feeding assistance. It is important to train nurses and nursing assistants to assess feeding problems in order to provide adequate assistance and maximize interactions to facilitate meals with PwD in long-term care facilities in Taiwanese.

## Supporting Information

S1 File Feeding Difficulty Index. (DOCX)

S2 File FDI_database_20150619.xls. (XLS)
