# Supplementary material for: Development and Psychometric Evaluation of the Chinese Feeding Difficulty Index (Ch-FDI) for People with Dementia
Source: PLoS One. 2015 Jul 21;10(7):e0133716. doi: 10.1371/journal.pone.0133716 (PMC4510378; doi:10.1371/journal.pone.0133716)
Supplement: S1 File — (DOCX) [file pone.0133716.s001.docx]

Appendix

**Feeding Difficulty Index**

This instrument assesses feeding difficulties in patients who require assistance with feeding. The items address the behaviors of the person during feeding by a caregiver. Although some of these behaviors may be seen in others during eating, the instrument is designed to be used with feeding difficulties while the caregiver is providing verbal or physical assistance with feeding. If the person has required assistance in feeding in the last 3 days, observe them during one entire meal and count the number of times a behavior is observed when food is offered by the caregiver. Each attempt to place the food in the mouth of the person is considered an offering.

| **Problem Behaviors** | | **offered** | | | |
| --- | --- | --- | --- | --- | --- |
|  |  | **0** | **1-2** | **3-5** | **≥6** |
| 1 | **Pushes or resists food offered by hand** |  |  |  |  |
| 2 | **Negative behavior toward feeder: pushes, hits, kicks, or throws objects at feeder** |  |  |  |  |
| 3 | **Inappropriate verbal statement toward feeder: negative statements about feeder, such as swearing at feeder** |  |  |  |  |
| 4 | **Turns head away or tilts head backward** |  |  |  |  |
| 5 | **Spits out the food** |  |  |  |  |
| 6 | **Does not open the mouth or bites the utensils when food is offered** |  |  |  |  |
| 7 | **Leaves the table** |  |  |  |  |
| 8 | **Cannot sit still: slipping or twisting body to affect eating** |  |  |  |  |
| 9 | **Does not start to eat for at least 1 minute when invited to do so** |  |  |  |  |
| 10 | **Becomes drowsy or falls asleep** |  |  |  |  |
| 11 | **Discontinues eating for over 1 minute** |  |  |  |  |
| 12 | **Distracted from eating by talking, looking around, or watching TV** |  |  |  |  |
| 13 | **Plays with food: does something with food but not eat it** |  |  |  |  |
| 14 | **Unable to successfully pick up food with utensil** |  |  |  |  |
| 15 | **Once food is on an eating utensil, unable to get the food effectively into the mouth** |  |  |  |  |
| 16 | **Uses hand to feed self** |  |  |  |  |
| 17 | **Once food is in the mouth, food dribbles out from the mouth** |  |  |  |  |
| 18 | **Continuously chews food or holds it in mouth but does not initiate swallowing** |  |  |  |  |
| 19 | **Chokes or gags on food** |  |  |  |  |

**Scoring of the Feeding Difficulty Index:**

The number of times a behavior is found with each offering of food is scored.

0 times per offering (0), 1 or 2 times per offering (1), 3~5 times per offering (2), 6 or more times (3).

The total score is the sum of the ratings that ranges from 0 to 72.
